# Supplementary material for: Splice-Junction-Based Mapping of Alternative Isoforms in the Human Proteome
Source: Cell Rep. Author manuscript; Available in PMC 2020 Jan 15. (PMC6961840; doi:10.1016/j.celrep.2019.11.026)
Supplement: 2 [file NIHMS1546469-supplement-2.zip › DF1/PXD006675/PulmonaryArtery/PulmonaryArtery_9_SORBS1_ESYLLQPSYPAKDATSSSAAQPEVIVVPLYLVNTDR.pdf]

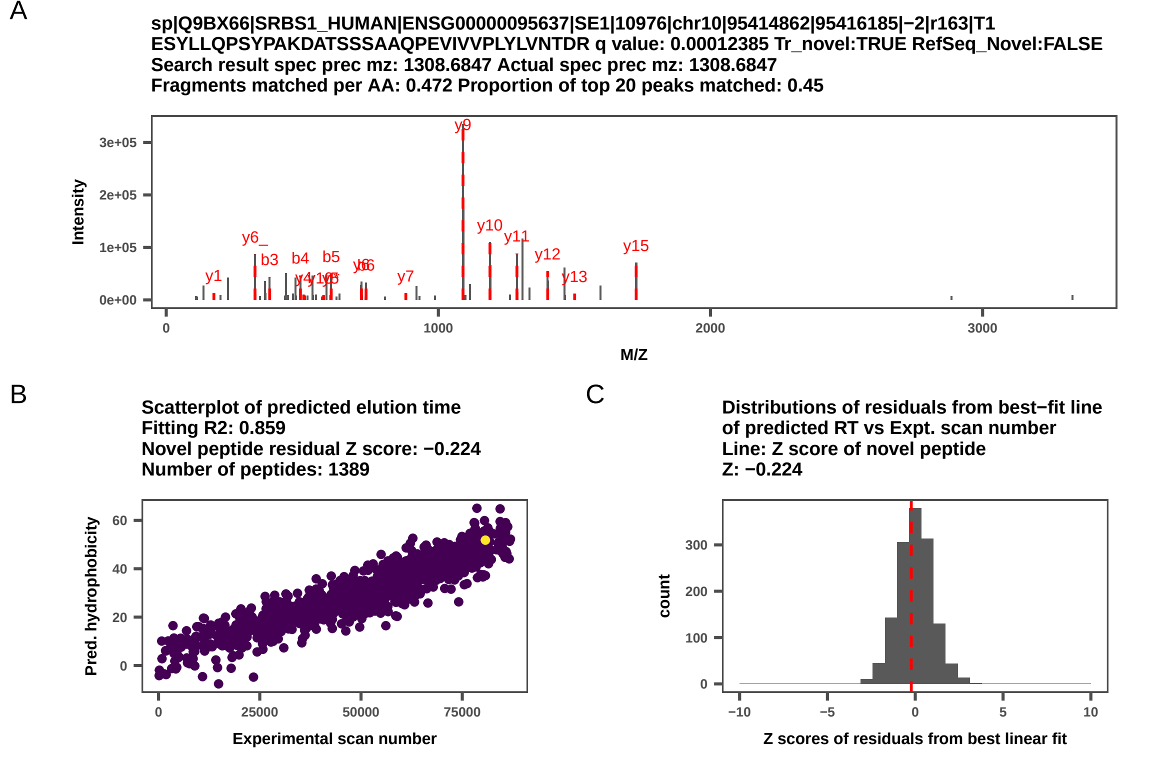
A
sp|Q9BX66|SRBS1_HUMAN|ENSG00000095637|SE1|10976|chr10|95414862|95416185|−2|r163|T1
ESYLLQPSYPAKDATSSSAAQPEVIVVPLYLVNTDR q value: 0.00012385 Tr_novel:TRUE RefSeq_Novel:FALSE
Search result spec prec mz: 1308.6847 Actual spec prec mz: 1308.6847
Fragments matched per AA: 0.472 Proportion of top 20 peaks matched: 0.45
Intensity
3e+05
2e+05
1e+05
0e+00
y9
y10
y11
y6_
b3
b4
b5
y1
y7
y12
y13
y15
M/Z
0
1000
2000
3000
B
Scatterplot of predicted elution time
Fitting R2: 0.859
Novel peptide residual Z score: −0.224
Number of peptides: 1389
Pred. hydrophobicity
60
40
20
0
Experimental scan number
0
25000
50000
75000
C
Distributions of residuals from best−fit line
of predicted RT vs Expt. scan number
Line: Z score of novel peptide
Z: −0.224
count
300
200
100
0
Z scores of residuals from best linear fit
−10
−5
0
5
10
